# A common genetic network underlies substance use disorders and disruptive or externalizing disorders

**DOI:** 10.1007/s00439-012-1164-4

**Published:** 2012-04-11

**Authors:** Mauricio Arcos-Burgos, Jorge I. Vélez, Benjamin D. Solomon, Maximilian Muenke

**Affiliations:** 1 Medical Genetics Branch, National Human Genome Research Institute, National Institutes of Health, Building 35, Room 1B-209, Bethesda, MD 20892-3717 USA; 2 Translational Genomics Group, Department of Translational Medicine, John Curtin School of Medical Research, ANU College of Medicine, Biology and Environment, The Australian National University, Canberra, ACT Australia

## Abstract

Here we summarize evidence obtained by our group during the last two decades, and contrasted it with a review of related data from the available literature to show that behavioral syndromes involving attention deficit/hyperactivity disorder (ADHD), externalizing disorders, and substance-use disorder (SUD) share similar signs and symptoms (i.e., have a biological basis as common syndromes), physiopathological and psychopathological mechanisms, and genetic factors. Furthermore, we will show that the same genetic variants harbored in different genes are associated with different syndromes and that non-linear interactions between genetic variants (epistasis) best explain phenotype severity, long-term outcome, and response to treatment. These data have been depicted in our studies by extended pedigrees, where ADHD, externalizing symptoms, and SUD segregate and co-segregate. Finally, we applied here a new formal network analysis using the set of significantly replicated genes that have been shown to be either associated and/or linked to ADHD, disruptive behaviors, and SUD in order to detect significantly enriched gene categories for protein and genetic interactions, pathways, co-expression, co-localization, and protein domain similarity. We found that networks related to pathways involved in axon guidance, regulation of synaptic transmission, and regulation of transmission of nerve impulse are overrepresented. In summary, we provide compiled evidence of complex networks of genotypes underlying a wide phenotype that involves SUD and externalizing disorders.

## Introduction

Complex phenotypes or traits result from a complicated interplay involving pleiotropy (production by a single gene of two or more effects), genetic heterogeneity (production of the same effect by two or more genes), epistasis (non-lineal genetic interactions), and environmental forces (Acosta et al. 2004). This type of trait exhibits a preferential familial clustering that cannot be explained by environmental causes alone, including shared cultural influences, and typically does not display patterns of Mendelian segregation (Acosta et al. 2004).

One of the best examples that demonstrate the way in which interacting effects can give rise to complex phenotypes is autoimmune disease (AID). Any chronic condition initiated by loss of immunological tolerance to self-antigens is considered an AID (Anaya 2010), part of a heterogeneous group of disorders that afflict specific target organs or multiple organ systems. Even though specific epidemiological, clinical, and pathological features define different AIDs, they have a number of clinical signs and symptoms in common. They also share pathophysiological mechanisms and genetic factors. Furthermore, it is possible to find in a single patient more than one AID. More interestingly, one pedigree might include a cluster of many affected individuals with different but related phenotypes (Anaya 2010). Mutations in one gene might be associated with several AIDs (Jin et al. 2010; Spritz 2011) and variants in two genes might converge in a cooperative interaction (epistasis) to outline a complex AID phenotype, as described in membranous nephropathy (Stanescu et al. 2011). Autoimmune diseases preferentially tend to affect females (i.e., they demonstrate a gender bias), share similar causal environmental agents, and several AIDs might respond to the same pharmacological treatment (Anaya 2010).

**Fig. 1:**
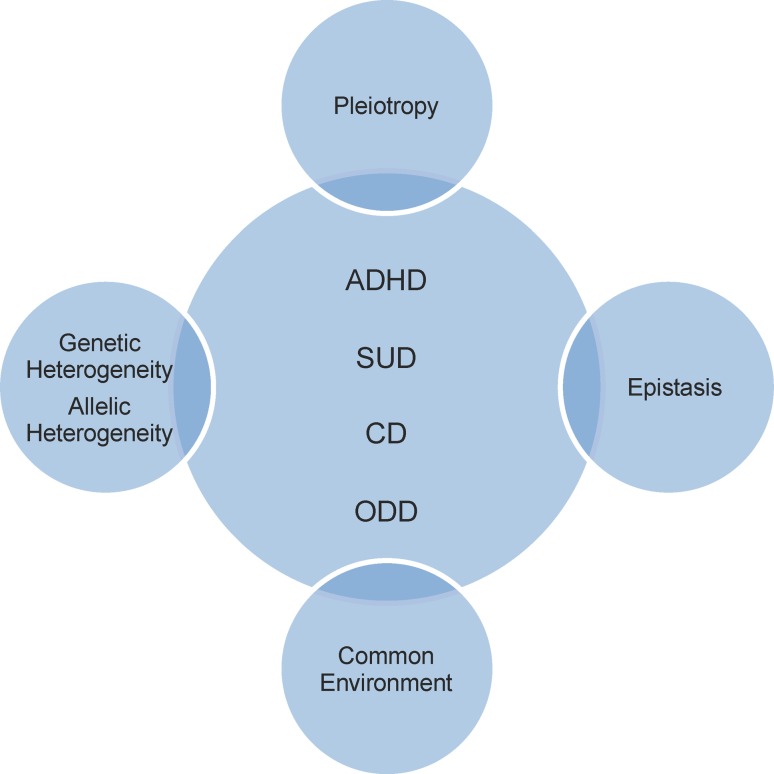
Physiopathological and psychopathological mechanisms, and genetic factors shared by attention deficit hyperactivity disorder (*ADHD*), conduct disorder (*CD*), oppositional defiant disorder (*ODD*) and substance-use disorder (*SUD*)

Evidence derived from several studies by our group suggests that common mental disorders of childhood and adolescence form a similar picture to that of the AIDs. The Diagnostic and Statistical Manual of Mental Disorders (DSM-IV) includes in its classification attention deficit/hyperactivity disorder (ADHD), disruptive behavior disorders including conduct disorder (CD) and oppositional defiant disorder (ODD), as well as substance-use disorder (SUD). In the following sections, we will present data that support our model proposing that these constructs, grouped under the label “externalizing disorders”, can be considered to be related syndromes, and that they share common physiopathological and psychopathological mechanisms, with evidence for common underlying genetic factors (Fig. 1) (Acosta et al. 2004; Arcos-Burgos et al. 2004a, 2010; Elia et al. 2009; Jain et al. 2011; Martinez et al. 2011; Pineda et al. 2011; Ribases et al. 2011).

**Fig. 2:**
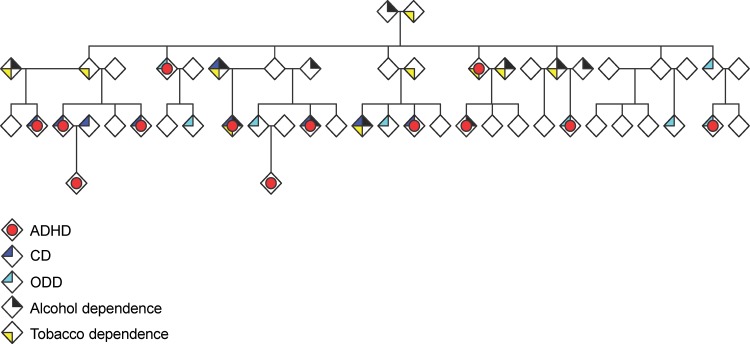
An extended pedigree demonstrating ADHD, externalizing symptoms, and associated conditions including nicotine, dependence and alcohol abuse and/or dependence. With modifications from Palacio et al. (2004)

Furthermore, we show that same genetic variants, harbored at different genes (loci), may be associated with different syndromes (Arcos-Burgos and Muenke 2010; Jain et al. 2007) and, in particular cases, non-linear interactions between these variants (epistasis) correlate with the specific phenotype, the severity of that phenotype, the long-term clinical outcome, and response to treatment (Jain et al. 2011). In addition, we will demonstrate that in extended pedigrees, ADHD, externalizing symptoms, and SUD can all be present simultaneously in one individual, and also, that different members of a single pedigree can exhibit variable combinations of these syndromes (Fig. 2). Finally, by applying formal network analysis, we will show that these apparently unrelated genes are overrepresented by statistically significant gene ontology (GO) networks related to pathways such as those that involve axon guidance, regulation of synaptic transmission, and regulation of transmission of nerve impulse.

## Definitions and background

Attention deficit/hyperactivity disorder is the most common neurodevelopmental behavioral disorder of childhood (Castellanos and Tannock 2002), and is characterized by elevated levels of inattention and/or hyperactive or impulsive behaviors that cause significant impairment (American Psychiatric Association 2000). Attention deficit/hyperactivity disorder affects 10 % of children and adolescents in the US (Visser et al. 2010); affected individuals are at increased risk for poor educational achievement, low income, underemployment, legal difficulties, and impaired social relationships (Arcos-Burgos et al. 2010). A conservative estimate of the annual societal cost of ADHD in childhood and adolescence is $ 42.5 billion in the US alone (Pelham et al. 2007).

Other externalizing disorders of childhood besides ADHD include CD, ODD, and juvenile bipolar disorder. Although ADHD can occur as a single disorder in a minority of diagnosed individuals, it generally co-occurs with other disruptive behaviors disorders and with SUD (Palacio et al. 2004).

Substance-use disorder, a term that includes substance abuse and dependence, is characterized by compulsive drug seeking and drug use even in the face of severe adverse consequences. The World Health Organization estimates that there are at least 2 billion alcohol users, more than 1 billion tobacco users, and almost 185 million illicit drug users worldwide (Li and Burmeister 2009). Vulnerability to addictions is a complex trait with strong genetic influences that are largely shared by abusers of different legal and illegal addictive substances (Karkowski et al. 2000; Liu et al. 2006; True et al. 1999; Tsuang et al. 1998; Uhl et al. 2001).

*ADHD plus SUD*: Individuals with ADHD are at increased risk for SUD (Bukstein 2008; Molina BSGM 1999; Szobot et al. 2007). Children diagnosed with ADHD monitored during the transition into adolescence exhibited higher rates of alcohol, tobacco, and psychoactive drug use than control groups of children without ADHD (Biederman et al. 1995; Molina and Pelham 2003). The lifetime risk for SUD is approximately 50 % in subjects with childhood ADHD that persists into adulthood (Biederman et al. 1995). Similarly, a high ADHD prevalence is found in adolescents with SUD (DeMilio 1989; Horner and Scheibe 1997; Kuperman et al. 2001). Also, it affects SUD prognosis: ADHD is associated with both earlier and more frequent alcohol-related relapses (Ercan et al. 2003) and lower likelihood of cannabis treatment completion (White et al. 2004).

Regardless of the type of substance, longitudinal studies demonstrate that ADHD onset precedes SUD, suggesting that the psychopathology of ADHD is not typically secondary to SUD (Wilens and Biederman 2006). As ADHD precedes SUD, it is reasonable to hypothesize that timely diagnosis and treatment of ADHD may reduce the occurrence and/or severity of SUD. Indeed, in a recent meta-analysis of six studies in which children with ADHD were treated with stimulants, the most common pharmacotherapy, SUD was less common in adolescence in treated versus non-pharmacologically treated children (Wilens et al. 2003).

Though the basis of the connection remains unclear, several theories have been proposed to explain the increased risk for SUD in patients with ADHD. Genetically mediated personality traits, such as novelty seeking and impulsivity, are common to ADHD and SUD, and they may provide a link to common neurologic substrates (Chambers et al. 2003; Wilens and Biederman 2006). It has also been proposed that patients with ADHD use addictive substances in an attempt to self-medicate symptoms of the disorder (Khantzian 1997), and that the poor judgment and impulsivity associated with ADHD contribute to the development of dependence (Wilens and Biederman 2006).

## A proposed genetic network underlying ADHD, CD, ODD, and SUD

### Clustering of ADHD, CD, ODD, and SUD in pedigrees

In 18 extended multigenerational families recruited from the genetically isolated Paisa community in Colombia, we found that a high proportion of individuals were affected with ADHD (32.8 %). These families also had high rates of CD (50 %; OR = 11.5, 95 % CI = 6.4–20.9), ODD (25.4 %; OR = 2.7, 95 % CI = 1.5–4.8), and associated conditions, including nicotine, dependence and alcohol abuse and/or dependence (Fig. 2) (Palacio et al. 2004), which were often comorbid with ADHD.

### Linkage studies of ADHD

**Fig. 3:**
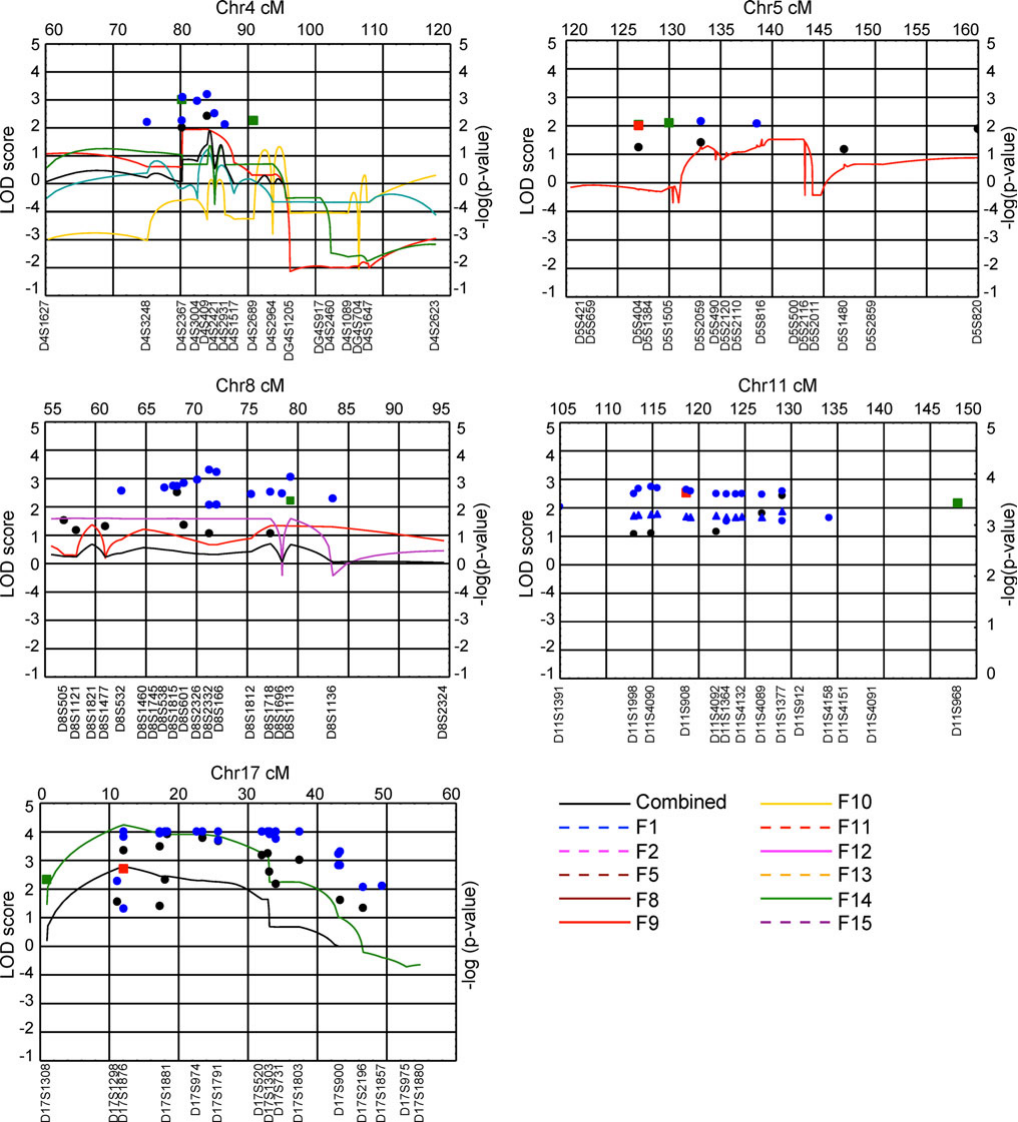
Model-based and model-free linkage analyses in extended and multigenerational Paisa families found evidence of linkage to markers at chromosomes 4q13.2, 5q33.3, 8q11.23, 11q22, and 17p11. These results were compatible with the presence of epistasis and pleiotropy in replication studies, suggesting that these loci harbor ADHD susceptibility genes. With modifications from Arcos-Burgos et al. (2004b)

We applied model-based and model-free linkage analyses and the pedigree disequilibrium test (PDT)(Martin et al. 2000) to genome-wide scan genotype data obtained for the Paisa families and found evidence of linkage to markers at chromosomes 4q13.2, 5q33.3, 8q11.23, 11q22, and 17p11 in individual families (Arcos-Burgos et al. 2004b). Fine mapping of these regions in the combined families resulted in significant linkage at chromosomes 4q13.2 (two-point allele sharing LOD score from LODPAL = 4.44 at D4S3248), 5q33.3 (two-point allele sharing LOD score from LODPAL = 8.22 at D5S490), 11q22 (two-point allele sharing LOD score from LODPAL = 5.77 at D11S1998, multipoint NPL −log(*P*) = 5.49 at ~128 cM) and 17p11 (multipoint NPL −log(*P*) >12 at ~12 cM, multipoint MLS = 2.48 (α = 0.10) at ~12 cM, two-point allele sharing LOD score from LODPAL = 3.73 at D17S1159). In addition, suggestive linkage at chromosome 8q11.23 was found (combined two-point NPL −log(*P*) >3.0 at D8S2332) (Arcos-Burgos et al. 2004b). Several of these regions were novel (4q13.2, 5q33.3, and 8q11.23) while others strongly replicated published loci (11q22 and 17p11). In summary, we identified linkage to the same genomic regions in different pedigrees, and to several genomic regions in specific pedigrees. These findings are compatible with the effects of epistasis and pleiotropy, respectively. The strong concordance between different analytical methods of linkage and the replication of data between two independent studies suggest that these loci harbor ADHD susceptibility genes (Fig. 3) (Arcos-Burgos et al. 2004b).

### Linkage and association commonalities of ADHD, CD, ODD, and SUD

**Fig. 4:**
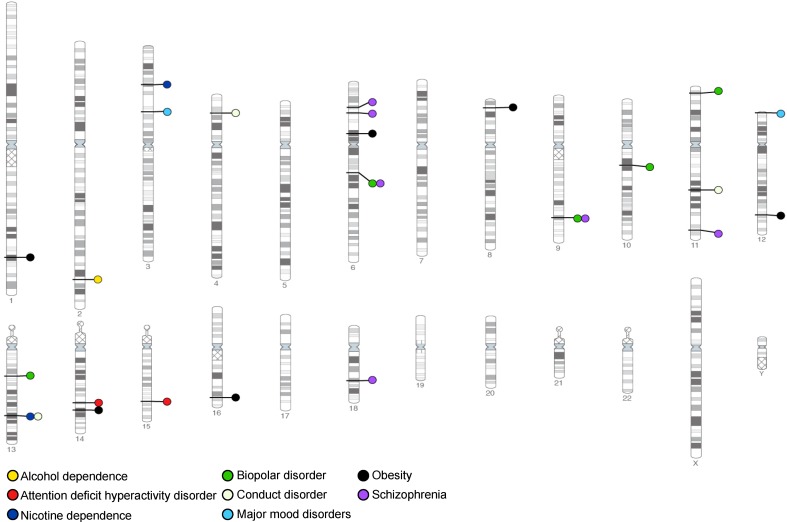
Linkage- and association-based genome scans identified loci containing common allelic variants contributing to SUD and to some psychiatric conditions

Both linkage- and association-based genome scans have been conducted for specific drug dependencies as well as the vulnerability to addiction. While each study has identified many different loci, these data converge to likely represent true contributions of common allelic variants to polygenic models of genetic vulnerability to polysubstance abuse, and to some psychiatric conditions that share common manifestations (Fig. 4).

In the extended and multigenerational Paisa pedigrees segregating ADHD comorbidly with ODD, CD, alcohol abuse/dependence, and nicotine dependence, we found that ADHD co-segregates with disruptive behaviors as a phenotypically variable trait, as evidenced by highly significant pair-wise linkages among ADHD and ODD (LOD = 14.19), ADHD and CD (LOD = 5.34), ODD and CD (LOD = 6.68), and CD and alcohol abuse/dependence (LOD = 3.98). In addition, we found evidence of linkage for comorbid ADHD phenotypes to loci at 8q24, 2p21–22.3, 5p13.1–p13.3, 12p11.23–13.3, 8q15, 11q22, and 14q21.1–22.2, suggesting that these patterns of cosegregation of ADHD with disruptive behavior and substance abuse comorbidities, and the linkage to similar regions, were indicative of a common major genetic cause (Jain et al. 2007). As additional evidence, some of these regions were highlighted by other studies, particularly the 11q22 chromosomal region that harbors the *NCAM1*, *TTC12*, *ANKK1*, and *DRD2* genes (Table 1).

**Table 1:** Results from GWAS and candidate gene approaches showing replicated genes associated with ADHD and SUD

Trait	Chr.	Marker	*P* value	Gene region	Type of study	Reference
ADHD	2	rs2556378	8.4 × 10^−7^	*BCL11A*	GWAS case–control	Hinney et al. (2011)
ADHD	11	rs5016282	1.8 × 10^−6^	*GRM5*	GWAS case–control	Hinney et al. (2011)
ADHD	16	rs8045006	2.3 × 10^−5^	*CDH13*	GWAS case–control	Neale et al. (2010)
Smoking behavior	11	rs10502172	9.1 × 10^−6^	*TTC12*-*ANKK1*-*DRD2*	Candidate gene case–control association	Ducci et al. (2011)
Smoking behavior	15	rs1051730,	1.1 × 10^−5^	*CHRNA3*	Candidate gene case–control association	Ducci et al. (2011)
Tobacco smoking	11	rs2303380–rs4938015–rs11604671	4.3 × 10^−2^	*TTC12*-*ANKK1*-*DRD2*	Candidate gene case–control association, black and white populations	David et al. (2010)
Alcohol dependence	11	rs1893699–rs723077	2.1 × 10^−4^	*NCAM1, TTC12, ANKK1, DRD2*	Candidate gene case–control and family-based association	Yang et al. (2007, 2008)
Nicotine dependence	11	rs2303380–rs4938012–rs4938015–rs11604671	1.0 × 10^−8^	*NCAM1*-*TTC12*-*ANKK1*-*DRD2*	Candidate gene case–control association	Gelernter et al. (2006)

### Evidence of linkage and association of ADHD, CD, ODD, and SUD to *LPHN3*

**Fig. 5:**
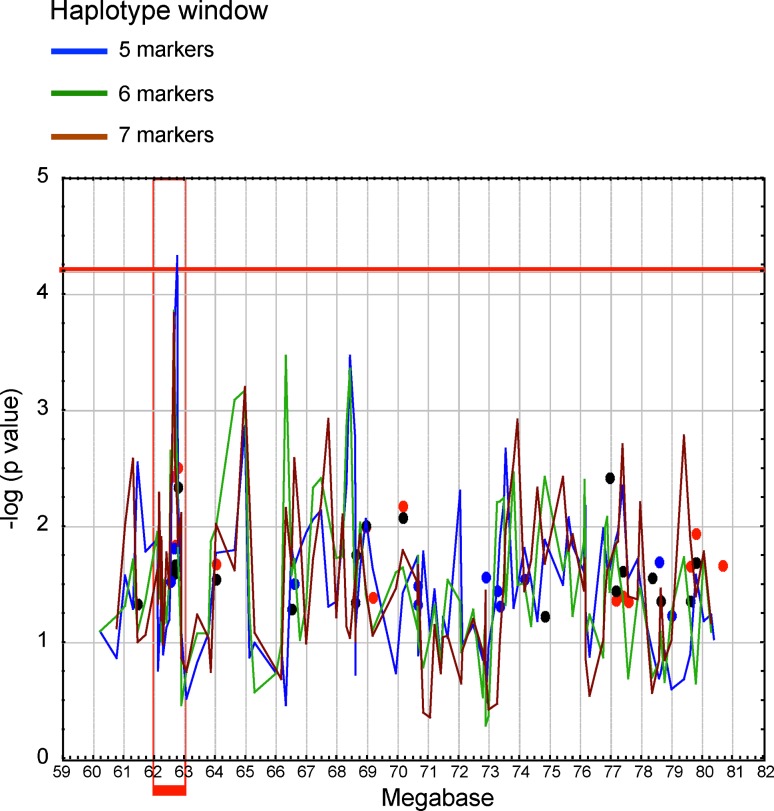
ADHD mapping by LD using cladistic analyses with closely spaced SNP markers across the critical region in 137 additional nuclear families reveals an area of association between 62.4 and 62.7 Mb in chromosome 4 (*in red*). With modifications from Arcos-Burgos et al. (2010)

Using the integration of statistical and functional approaches, we discovered a novel gene that segregates with ADHD and contributes to disease susceptibility; this gene is harbored in the chromosome 4q region previously found to be linked to ADHD (Fig. 5). The application of fine mapping to these linked families sharpened the linkage signal and revealed new meiotic recombination events in individuals with ADHD, which further narrowed the minimal critical region with the gene to ~20 Mb (Arcos-Burgos et al. 2010) (Fig. 5). Fine-scale genetic association was conducted in nuclear and large multigenerational Paisa families. Areas of interest included those that were gene-rich or that included potential candidate genes, and were covered at a higher density (Arcos-Burgos et al. 2010). Using family-based association strategies and haplotype-based cladistic analysis, a significant area of association with ADHD was then defined by the single nucleotide polymorphic (SNP) markers rs1901223 and rs1355368 (*P* = 3.1 × 10^−3^, marker-based; *P* = 2.7 × 10^−5^, haplotype based) (Arcos-Burgos et al. 2010) (Fig. 5).

**Fig. 6:**
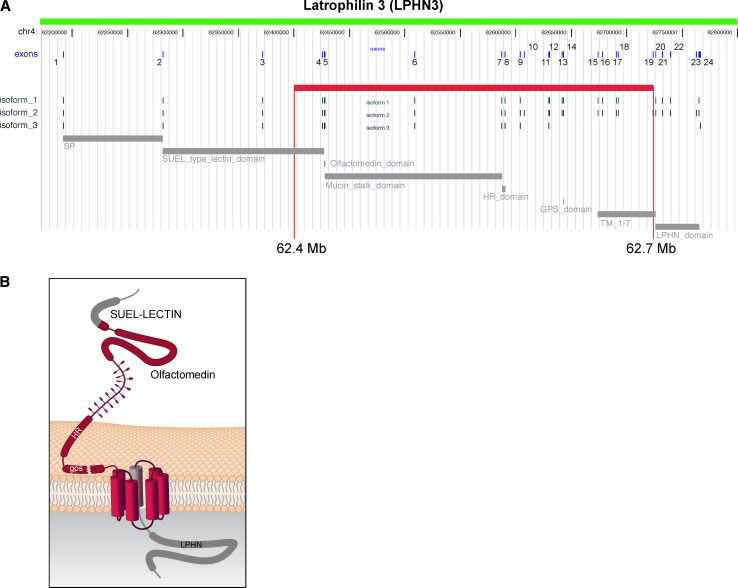
**a** The susceptibility haplotype encompasses exons 4–19 of LPHN3, and contains important functional domains and variable splicing sites for isoforms of the gene. There are not other genes annotated over the region spanned by the susceptibility haplotype. **b** General structure of latrophilins. The long extracellular region contains four domains: a SUEL LECTIN domain, a region homologous to olfactomedins and myocilin, a homology region (HR) with BAI1-3, and a cysteine-rich GPCR proteolysis site (*gps*). With modifications from Arcos-Burgos et al. (2010)

The region of association was located at 62.4–62.7 Mb (UCSC coordinates) on 4q within exons 4 through 19 of the *Latrophilin3* gene (*LPHN3*) (Arcos-Burgos et al. 2010). Latrophilin3 is a member of the latrophilin (LPHN) subfamily of G-protein coupled receptors (GPCRs). Genes encoding the GPCRs, such as *DRD4* and *DRD5*, have also been shown to be associated with ADHD (Arcos-Burgos et al. 2010) (Fig. 6a).

Latrophilins have seven transmembrane regions, a long N-terminal extracellular sequences containing a 19-amino acid signal peptide (GPCR proteolytic site, GPS domain), and a serine/threonine-rich glycosylation region (Martinez et al. 2011). Latrophilins 1 and 2 serve as receptors for α-latrotoxin, a component of the venom of the black widow spider (Martinez et al. 2011); this component interacts with neuronal GPCRs to stimulate exocytosis of GABA-containing presynaptic vesicles. GABA is an inhibitory neurotransmitter. As LPHN3 is the most brain-specific latrophilin, it suggests a possible role in ADHD (Martinez et al. 2011) (Fig. 6b).

Once the study of Paisa families identified a specific region of the *LPHN3* gene associated with ADHD, fine mapping was performed. This allowed us to precisely pinpoint variants in the DNA code that may alter function. In order to validate these findings, we pursued replication in additional samples from Colombia, Germany, Norway, Spain, and in two separate US populations. These meta-analyses showed evidence for a significant homogeneous genetic effect for three of the top associated markers inside the *LPHN3* gene (Arcos-Burgos et al. 2010). Replication of the association of *LPHN3* with ADHD in adults was also found in an independent sample from Spain (Ribases et al. 2010).

Combined efforts of this collaboration revealed potentially functional sequences within the *LPHN3* gene that may be considered targets for future studies. These findings are thus not only a step toward a better understanding of ADHD, but also offer an example of how multidisciplinary groups can interact to dissect causes of genetically complex human diseases (Arcos-Burgos et al. 2010).

In addition to the genetic studies, we carried out pathologic studies of brain specimens and neuroimaging studies. This showed that a key *LPHN3* variant of interest is expressed in brain regions related to attention and activity including the amygdala, caudate nucleus, cerebellum, and cerebral cortex. Importantly, the same variant associated with ADHD was also associated with response to stimulant medication (Arcos-Burgos et al. 2010). In other words, specific DNA sequence changes in the gene coding for LPHN3 were associated with and predicted whether or not a person with ADHD responded to and benefitted from treatment with stimulant medication.

*LPHN3* is thus an example of a locus where multiple independent psychiatric studies converge, and recapitulates earlier findings involving SUD. Initially, alcohol abuse was linked to Chromosome 4 in the area of *LPHN3* (Long et al. 1998). This study investigated a Southwestern American Indian tribe by genome-wide linkage analysis and reported linkage to marker D4S1645, which is less than 300 kb upstream of *LPHN3*. Another independent study utilized two-point linkage analysis in families with alcohol dependence to identify a nominally significant locus at D4S244 (Reich et al. 1998), which is ~5 Mb downstream of *LPHN3*. While its distance from LPHN3 makes this latter report less compelling, it nonetheless suggests that there is a locus on chromosome 4p related to alcohol dependence (Uhl 2004a, b).

Additional evidence is supplied by a third study that utilized whole genome association analysis to identify SNPs with significant allele frequency differences between abusers of illegal substances and control populations of both European-American (EA) and African-American (AA) ethnicities (Liu et al. 2006). This study identified three SNPs within *LPHN3* that are positive for association (Monte Carlo *P* = 5.89 × 10^−3^). This group utilized this information and replicated these as well as additional *LPHN3* SNPs in several populations including polysubstance abuser individuals from the National Institute on Drug Abuse (NIDA) (Bergen et al. 1999), samples from alcohol-dependent individuals from the Collaborative Studies on Genetics of Alcoholism (COGA) (Johnson et al. 2006; Liu et al. 2006), and samples from methamphetamine-dependent individuals from Japan and Taiwan (Uhl et al. 2008a, b).

We also provided evidence of epistasis underlying ADHD by screening for potential interactions between ADHD-linked loci, where we found a significant correlation between 4q and 11q (*P* < 1 × 10^−8^) (Jain et al. 2011). Conditioning on the *LPHN3* associated SNP, rs6551665, and using case–control and family-based designs, we detected interacting effects with a haplotype harbored on 11q (*P* < 5 × 10^−6^). This haplotype encompasses *Neural Cell Adhesion Molecule 1* (*NCAM1*), a gene with a fundamental role in neural development, the *Dopamine Receptor D2* (*DRD2*), a gene previously associated with ADHD, and the *Tetratricopeptide Repeat Domain 12* (*TTC12*), and *Ankyrin repeat and Kinase Domain Containing 1* (*ANKK1*), two genes associated with externalizing symptoms and nicotine dependence (David et al. 2010; Ducci et al. 2011; Gelernter et al. 2006; Yang et al. 2007, 2008) (Table 1).

Using three additional ADHD datasets that previously showed the association of ADHD with *LPHN3,* the *LPHN3*-11q interaction was replicated by formal meta-analysis (Jain et al. 2011). The effect of the interaction represents a two-fold increase from the original risk conferred by *LPHN3* to develop ADHD (OR = 2.46, 95 % CI = 1.63–3.70, *P* = 5.09 × 10^−6^). In addition, the *LPHN3*-11q interaction more significantly demonstrates the effect that the *LPHN3* susceptibility variant has on both ADHD response to stimulant medication and on brain metabolism (Jain et al. 2011). Furthermore, we demonstrated that this genetic interaction not only predicts ADHD severity but also long-term outcome, which is related to comorbidities (Acosta et al. 2011).

### Evidence of linkage and association of the *NCAM1*-*TTC12*-*ANKK1*-*DRD2* gene cluster to SUD

A prospective study of 4,762 finish subjects (52 % female) for whom smoking behaviors were ascertained at age 14 and 31 years, evaluated the developmental changes and the potential effect of genetic and non-genetic factors (Ducci et al. 2011). In this study, the A allele of rs1051730 SNP marker, located in the *Cholinergic Receptor alpha 3* (*CHRNA3*) gene, was found to have a similar effect-size at 14 years and 31 years and was more common in those described as heavy/regular smokers compared to those who did not smoke (*P* = 1.1 × 10^−5^).

Markers in the *TTC12*-*ANKK1*-*DRD2* genomic region have been also associated with smoking behavior, with rs10502172 (*P* = 9.1 × 10^−6^) being the most significantly associated marker. This marker is located within the *TTC12* gene; individuals with the G variant had 1.33 (95 % CI = 1.11–1.60) times higher risk of being smokers at age 14, and 1.14 (95 % CI = 1.02–1.28) times higher risk of being smokers at age 31 years. In adolescence, this variant also conferred increased risk of occasionally (OR = 1.12, 95 % CI = 1.12–1.48) smoking (Ducci et al. 2011). Furthermore, variants in the *ANKK1* (rs2734849) and *DRD2* (rs1076563) genes were also found to be significantly associated with smoking behavior.

In tobacco smoking, linkage studies and haplotype-based analyses on the *TTC12*-*ANKK1*-*DRD2* gene cluster have also elucidated genetic and sex-specific associations with smoking behavior (David et al. 2010). Seven SNPs within this gene cluster were tested using longitudinal data from 1993 to 1994 and 2004 to 2005 in 638 individuals (including 270 individuals of black and 368 of white ethnicity) from the Baltimore Epidemiologic Catchment Area (ECA) cohort study. Evidence was provided for sex-specific differences in smoking behavior. First, black male smokers with the GTG haplotype (defined by the markers rs2303380–rs4938015–rs11604671) were more likely to stop smoking 11 years later (55.6 % GTG vs. 22.0 % other haplotypes), in contrast with black women who had other haplotypes (20.8 % GTG vs. 24.0 % other haplotypes). Conversely, the same haplotype (vs. other haplotypes) was significantly associated with a lifetime history of daily smoking (OR = 1.6, 95 % CI = 1.1–2.4) and a higher prevalence of smoking initiation (77.6 % GTG vs. 57.0 % other haplotypes) in white individuals. Second, a different haplotype block was identified when comparing black and white individuals; this block consisted of one SNP within *TTC12* (rs2303380) and three in *ANKK1* (markers rs4938012, rs4938015, and rs11604671).

As in tobacco behavior, case/control- and family-based haplotype analyses with 43 SNPs within the *NCAM1*-*TTC12*-*ANKK1*-*DRD2* gene cluster have been performed in studies of alcohol dependence (AD) (Yang et al. 2007) and drug dependence (DD) (Yang et al. 2008). In the first study, two separate association studies of AD were conducted on 1,220 individuals of EA ancestry using family-based (488 individuals) and case–control (732 individuals, 43.4 % cases) designs (Yang et al. 2007). In this study, haplotypic variants within this gene cluster were associated with AD at *TTC12* (markers rs1893699–rs723077) in both the case–control and family-based samples, as well as variants around exon 12 of *NCAM1* and exons 2 and 5 of *ANKK1* only in the case–control sample. For DD, two separate association studies of AD with DD (AD + DD) and AD without DD (AD-only) in 1,090 individuals of EAs were performed using the aforementioned designs and the same genetic variants (Yang et al. 2008). More evidence was provided for the association of haplotypic variants in exon 3 of *TTC12*, exon 12 of *NCAM1*, and the two 3′-ends of *ANKK1* and *DRD2* as co-effectors of AD and DD.

It is intriguing that variants within the *NCAM1*-*TTC12*-*ANKK1*-*DRD2* gene cluster were also found to be strongly associated with nicotine dependence (ND) in two independent American populations, consisting of 1,615 individuals from AA and EA ancestry (Gelernter et al. 2006). These individuals were based on affected sibling pairs with cocaine or opioid dependence. Forty-three SNPs were genotyped (see above). Of these, four markers (rs4938012, rs4938013, rs4938015, and rs11604671) located in the *ANKK1* gene were significantly associated with ND in EA, and only the first two markers reached nominal significance in AA. In *TTC12,* the most statistically significant marker was rs2303380 (*P* < 0.0007 in AAs; *P* < 0.007 in EAs). Haplotype analyses performed on the most significantly associated markers (rs2303380, rs4938012, rs4938015, and rs11604671) demonstrated a significant association of this SNP combination with ND in the individual (*P* < 0.0003 for AAs; *P* < 0.0008 for EAs) and pooled (*P* < 0.000005) samples. Furthermore, the GATC haplotype was associated with ND (EAs: *P* < 0.00006; AAs: *P* < 0.0008; pooled: *P* < 0.0000001) showing strong evidence for association of the same haplotype with ND. In these two distinct populations, the AGCT and AGTC haplotypes were associated with decreased risk of ND (EAs: *P* < 0.001; AAs: *P* < 0.0009) (Gelernter et al. 2006).

### Evidence of a genetic network underlying ADHD, CD, ODD, and SUD

**Fig. 7:**
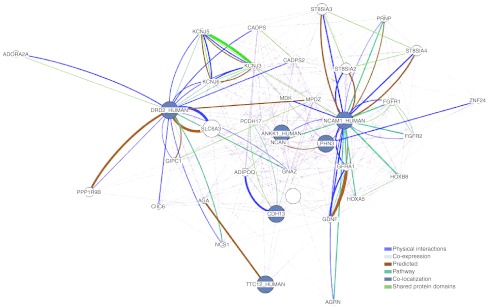
Results from a formal network analysis using the *ANKK1*, *TTC12*, *DRD2*, *NCAM1*, *LPHN3*, and *CDH13* genes in order to detect significantly enriched gene categories for protein and genetic interactions, pathways, co-expression, co-localization and protein domain similarity. These selected genes were significantly replicated as being either associated and/or linked to ADHD, disruptive behaviors and SUD. Networks related to pathways involved in processes such as axon guidance, regulation of synaptic transmission and regulation of transmission of nerve impulse were overrepresented. For more information see Tables 2 and 3

In order to contrast the main hypothesis of the manuscript, i.e., the existence of a network of genes underlying these complex phenotypes, we performed a formal network analysis with the *LPHN3*, *NCAM1*, *TTC12*, *ANKK1*, *DRD2*, and *CDH13* genes, a set of significantly replicated genes either associated and/or linked to ADHD, disruptive behaviors, and SUD (Table 1). In addition, in order to detect significantly enriched gene categories within this set of genes, we performed analyses with the GeneMANIA Pathway Analysis software (http://genemania.org/). GeneMANIA uses a large set of available functional association data for protein and genetic interactions, pathways, co-expression, co-localization, and protein domain similarity. We found additional genes in pathways (Table 2 and Fig. 7), as well as functions that frame these apparently unrelated genes to statistically significant gene ontology (GO) networks related to pathways, such as axon guidance, regulation of synaptic transmission, and regulation of transmission of nerve impulse, among others (Table 3).

**Table 2:** Genes overrepresented in gene ontology (GO) networks involving the *LPHN3, NCAM1*, *TTC12*, *ANKK1,* *DRD2, and CDH13* genes

Gene	Name
*SLC6A3*	Solute carrier family 6 (neurotransmitter transporter, dopamine), member 3
*ST8SIA3*	ST8 alpha-N-acetyl-neuraminide alpha-2,8-sialyltransferase 3
*ST8SIA2*	ST8 alpha-N-acetyl-neuraminide alpha-2,8-sialyltransferase 2
*ST8SIA4*	ST8 alpha-N-acetyl-neuraminide alpha-2,8-sialyltransferase 4
*PPP1R9B*	Protein phosphatase 1, regulatory (inhibitor) subunit 9B
*ADIPOQ*	Adiponectin, C1Q and collagen domain containing
*GFRA1*	GDNF family receptor alpha 1
*FGFR1*	Fibroblast growth factor receptor 1
*ADORA2A*	Adenosine A2a receptor
*GDNF*	Glial cell derived neurotrophic factor
*PRNP*	Prion protein
*NCS1*	Neuronal calcium sensor 1
*AGA*	Aspartylglucosaminidase
*CLIC6*	Chloride intracellular channel 6
*AGRN*	Agrin
*GIPC1*	GIPC PDZ domain containing family, member 1
*MDK*	Midkine (neurite growth-promoting factor 2)
*HOXB8*	Homeobox B8
*NCAN*	Neurocan
*KCNJ3*	Potassium inwardly-rectifying channel, subfamily J, member 3
*ZNF24*	Zinc finger protein 24
*FGFR2*	Fibroblast growth factor receptor 2
*KCNJ5*	Potassium inwardly-rectifying channel, subfamily J, member 5
*CADPS*	Ca^++^-dependent secretion activator
*KCNJ6*	Potassium inwardly-rectifying channel, subfamily J, member 6
*MPDZ*	Multiple PDZ domain protein
*CADPS2*	Ca^++^-dependent secretion activator 2
*GNAZ*	Guanine nucleotide binding protein (G protein), alpha z polypeptide
*PCDH17*	Protocadherin 17
*HOXA5*	Homeobox A5

**Table 3:** Gene ontology (GO) networks overrepresented when considering the *LPHN3, NCAM1*, *TTC12*, *ANKK1,* *DRD2, and CDH13* genes

GO ID	Description	*q* value
0007411	Axon guidance	1.90 × 10^−5^
0050804	Regulation of synaptic transmission	3.97 × 10^−3^
0005242	Inward rectifier potassium channel activity	4.32 × 10^−3^
0051969	Regulation of transmission of nerve impulse	4.32 × 10^−3^
0031644	Regulation of neurological system process	4.32 × 10^−3^
0008373	Sialyltransferase activity	4.68 × 10^−3^
0051937	Catecholamine transport	5.01 × 10^−3^
0015844	Monoamine transport	1.18 × 10^−2^
0015850	Organic alcohol transport	2.79 × 10^−2^
0034705	Potassium channel complex	3.59 × 10^−2^
0008076	Voltage-gated potassium channel complex	3.59 × 10^−2^
0005249	Voltage-gated potassium channel activity	3.59 × 10^−2^

These results are compatible with other network analyses that used the most highly ADHD-associated genes, which found that they encode proteins involved in directed neurite outgrowth (Poelmans et al. 2011). With this additional data, we show that common genetic variants harbored in the *LPHN3*, *NCAM1*, *TTC12*, *ANKK1*, *DRD2*, and *CDH13* genes, known to predispose to ADHD and to disruptive behavior symptoms present in ODD, CD, and SUD, are overrepresented in a network of genes ontologically linked to neurodevelopment. Future deep sequencing of the genomic regions harboring these variants will allow us to define true functional causal mutations. Identified common and rare genetic variants, together with clinical covariate information, plus data of brain function provided by fMRI, will in the future provide a predictive framework suitable to be used in clinical assessment and prevention of these common behavioral disorders.
